# Angiogenesis and chronic kidney disease

**DOI:** 10.1186/1755-1536-3-13

**Published:** 2010-08-05

**Authors:** Yohei Maeshima, Hirofumi Makino

**Affiliations:** 1 Department of Medicine and Clinical Science, Okayama University Graduate School of Medicine, Dentistry and Pharmaceutical Sciences, Okayama, Japan

## Abstract

The number of patients requiring renal replacement therapy due to end-stage renal disease (ESRD) is increasing worldwide. The prevalence of chronic kidney disease (CKD), and the importance of CKD as a risk factor in development of ESRD and in complicating cardiovascular disease (CVD) have been confirmed. In recent years, the involvement of angiogenesis-related factors in the progression of CKD has been studied, and the potential therapeutic effects on CKD of modulating these factors have been identified. Vascular endothelial growth factor (VEGF)-A, a potent pro-angiogenic factor, is involved in the development of the kidney, in maintenance of the glomerular capillary structure and filtration barrier, and in the renal repair process after injury. VEGF-A is also involved in the development of early diabetic nephropathy, demonstrated by the therapeutic effects of anti-VEGF-A antibody. Angiopoietin (Ang)-1 induces the maturation of newly formed blood vessels, and the therapeutic effects of Ang-1 in diabetic nephropathy have been described. In experimental models of diabetic nephropathy, the therapeutic effects of angiogenesis inhibitors, including angiostatin, endostatin and tumstatin peptides, the isocoumarin NM-3, and vasohibin-1, have been reported.

Further analysis of the involvement of angiogenesis-related factors in the development of CKD is required. Determining the disease stage at which therapy is most effective and developing an effective drug delivery system targeting the kidney will be essential for pro-or anti-angiogenic strategies for patients with CKD.

## Introduction

The number of patients with chronic kidney disease (CKD) progressing to end-stage renal disease (ESRD) and requiring renal replacement therapy is increasing worldwide. CKD currently affects over 20 million adults in the USA and over 13 million adults in Japan [1,2]. Of the various renal disorders predisposing to CKD, including glomerulonephritis and hypertensive nephrosclerosis, diabetic nephropathy is the most frequent cause of ESRD development.

Angiogenesis - the development of new blood vessels from pre-existing ones - is involved in physiological events and in pathological disorders including tumor growth and metastasis, proliferative retinopathy, rheumatoid arthritis, psoriasis and neointimal formation [3]. Angiogenesis is controlled by the balance between pro-angiogenic and anti-angiogenic factors. Angiogenesis-associated factors are involved in the development of the kidney [4-6]. Recent experimental studies have demonstrated the involvement of an imbalance of angiogenesis-related factors in the progression of CKD [7-13], and the potential therapeutic effects on CKD of modulating these factors have been identified [14-22]. Vascular endothelial growth factor (VEGF)-A, a potent pro-angiogenic factor, is involved in the development of the kidney [4,5], and also plays an important role in maintaining the glomerular capillary structure and in the repair process following injuries of glomerular endothelial cells and peritubular capillaries (PTC) [14,15,17]. Physiological levels of VEGF-A are also required for maintenance of the glomerular filtration barrier [23]. In the early stages of diabetic nephropathy, increases in the number of glomerular capillaries and in the glomerular levels of VEGF-A and its receptor VEGFR-2 are observed [10,24]. The therapeutic effects of anti-VEGF-A strategies and anti-angiogenic factors in diabetic nephropathy have been reported [19-21,25-30].

In this review, the biological function of angiogenesis-associated factors in CKD and the therapeutic potential of modulating these factors are summarized.

## Role of VEGF-A, VEGFR and other angiogenic growth factors in healthy kidney

A number of angiogenic growth factors are involved in the development of the kidney and in the maintenance of glomerular structures and the glomerular filtration barrier function in adults. Physiological levels of angiogenic factors such as VEGF-A and angiopoietin (Ang)-1 are crucial for maintaining intact glomerular structures and glomerular filtration function. In some cases, proteinuria and endothelial dysfunction may be complicated by excessive inhibition of these factors (for example, treatment with anti-VEGF antibodies in patients with malignancy). In this section, the roles of VEGF-A, VEGFRs and other angiogenic factors including angiopoietins in the healthy kidney are reviewed (Table 1).

**Table 1:** Expression and biological roles of angiogenic factors in health and CKD

	Healthy kidney	Human CKD (non-diabetic)	Diabetic nephropathy	Therapeutic effects
VEGF-A	Podocyte (h, m, r), TEC (h)	Decreased (renal)	Decreased (h), Increased (h, m, r)	Anti-Thy1 nephritis (r), anti-GBM nephritis (r), thrombotic microangiopathy (r), remnant kidney (r)
VEGFR-1	GEnC (h, m), PTC (h)	MC (Mesangio-proliferative GN)	NE	NE
VEGFR-2	GEnC (h, m), podocyte (m), MC (h), PTC (h)	MC (Mesangio-proliferative GN)	Increased (r)	NE
sVEGFR-1	NE	Increased: pre-eclampsia, CKD (serum)	NE	Diabetic nephropathy (m)
Neuropilin-1	GEnC (h), podocyte (h), MC (h), TEC (m)	NE	NE	NE
Ang-1	podocyte (h, m)	Decreased: CKD (serum)	Decreased (r; renal)	Obstructive uropathy (m), diabetic nephropathy (m)
Ang-2	TEC (m)	Increased: CKD (serum)	Increased (h; serum), increased (r, m; renal)	NE

Ang-1 = angiopoietin-1; Ang-2 = angiopoietin-2; CKD = chronic kidney disease; GEnC = glomerular endothelial cells; GBM = glomerular basement membrane; GN = glomerulonephritis; h = human; m = mouse; MC = mesangial cell; NE = not examined; PTC = peritubular capillaries; r = rat; TEC = tubular epithelial cells.

### VEGF-A

The role of VEGF-A in regulating angiogenesis has been intensively investigated. VEGF-A signaling is crucially involved in physiological and pathological angiogenesis (for example tumor growth) [31]. The VEGF gene family consists of VEGF-A, VEGF-B, VEGF-C and placental growth factor (PlGF) [31]. VEGF-A is a key regulator of blood vessel growth, whereas VEGF-C and VEGF-D are involved in regulating lymphatic angiogenesis [32]). Inactivation of a single *Vegf *allele in mice resulted in embryonic lethality, owing to multiple developmental anomalies including defective vascularization in several organs, suggesting an essential role of VEGF in embryonic vasculogenesis and angiogenesis [33,34]. VEGF-A induces the proliferation of endothelial cells *in vitro *[31], induces angiogenic response *in vivo *[35], and also induces the survival of endothelial cells [36]. VEGF-A affects vascular permeability [37] and promotes monocyte chemotaxis [38], leading to inflammation and other pathological consequences. VEGF-A also induces endothelium-dependent vasodilatation, in association with endothelium-derived nitric oxide (NO) [39]. Consistent with its role in regulating vascular permeability, VEGF-A induces fenestration of endothelial cells in some vascular beds, including glomerular endothelial cells [40].

Several different splice variant isoforms of VEGF-A (VEGF_121_, VEGF_165_, VEGF_165b_, VEGF_189_, VEGF_206_) have been reported [41-43]. The properties of native VEGF-A (45 kDa) closely correspond to those of predominant isoform, VEGF_165 _[44]. Expression of VEGF-A mRNA is induced under hypoxic conditions in endothelial cells, mediated through hypoxia-inducible factor (HIF)-1 [45]. In addition, growth factors such as transforming growth factor (TGF)-α, TGF-β, [Au Query: Please check all symbols in the paper - they did not convert properly *Answer: OK*.] insulin-like growth factor (IGF)-1, platelet-derived growth factor (PDGF) and basic fibroblast growth factor upregulate the expression of VEGF-A mRNA [31].

VEGF-A binds to the tyrosine kinase receptors VEGFR-1 (also known as Flt-1) and VEGFR-2 (also known as KDR/Flk-1) [31], and VEGF-C and VEGF-D bind to VEGFR-3 [32]. VEGF-A also binds to a family of co-receptors, the neuropilins [46]. VEGF_165b _antagonizes the effects of VEGF-A, and exerts anti-angiogenic effects [43].

VEGF-A is constitutively expressed in the human kidney, primarily in the glomerular visceral epithelial cells (podocytes) and in tubular epithelial cells in the outer medulla and medullary rays, more commonly observed in distal tubules and collecting ducts than in proximal tubules [47,48]. VEGF-A is also expressed in mesangial cells *in vitro *[49] and in disease models, but not in mesangial cells of the healthy human kidney [47]. Podocyte-specific heterozygous deletion of VEGF-A in mice resulted in proteinuria and glomerular endothelial injury similar to pre-eclampsia, and podocyte-specific overexpression of VEGF-A led to collapsing glomerulopathy, similar to the lesions observed in HIV-associated nephropathy [23]. Therefore, physiological levels of VEGF-A are required for maintenance of the glomerular filtration barrier [23]. In newborn mice, blockade of VEGF-A with antibodies resulted in abnormal glomeruli lacking capillary tufts and in decreased nephron number, suggesting a pivotal role for VEGF-A in the development of glomerular vascular structures [4]. Transgenic rabbits expressing human VEGF_165 _in both the kidney and liver exhibited proteinuria and progressive renal dysfunction, with glomerular hypertrophy and proliferation of mesangial and glomerular endothelial cells [50]. At a later stage, these transgenic rabbits developed microaneurysms, leading to glomerulosclerosis, glomerular cyst formation and tubulointerstitial injuries [50]. More recently, interstitial fibrosis, tubular cyst formation, proliferative enlargement of glomerular capillaries and mesangial proliferation were observed in transgenic adult mice with tubule-specific overexpression of VEGF-A [51]. Veron *et al*. induced podocyte-specific overexpression of VEGF-A in adult mice. The authors observed proteinuria, glomerulomegaly, thickening of the glomerular basement membrane (GBM), mesangial expansion, and podocyte injuries in these mice, similar to the findings observed in diabetic nephropathy [52].

In patients with cancer treated with bevacizumab, a humanized monoclonal anti-VEGF-A antibody, thrombotic microangiopathy developed as a complication. Podocyte-specific deletion of VEGF-A in adult mice produced similar histological lesions [53]. Similar effects of anti-VEGF-A strategies in healthy mice, leading to endothelial dysfunction and proteinuria have been reported [54]. Collectively, the physiological level of VEGF-A is important for the maintenance of renal structures and of an intact filtration barrier in the developing and adult kidney.

VEGF_165b_, an inhibitory splice variant of VEGF-A, is produced by cultured human podocytes [55]. Denys-Drash syndrome is a rare urogenital disorder characterized by male pseudohermaphroditism. It carries a high risk for Wilms' tumor and diffuse mesangial sclerosis, caused by mutations affecting the zinc finger structure of the Wilms' tumor protein 1 [56]. Podocytes obtained from patients with Denys-Drash syndrome express high levels of VEGF_165_, but lack VEGF_165b_, potentially associated with the development of glomerulosclerosis [57].

### VEGFR-1 (Flt-1)

VEGF-B and PlGF do not bind to VEGFR-2, but like VEGF-A, they do bind to VEGFR-1. Upon binding to VEGF-A, VEGFR-1 undergoes weak tyrosine autophosphorylation [58]. A soluble extracellular domain of VEGFR-1 (sFlt-1) serves as a VEGF inhibitor [59]. VEGFR-1^-/- ^mice die *in utero *at embryonic days (E)8.5-9.5, primarily due to excessive proliferation of angioblasts, suggesting that VEGFR-1 negatively regulates the activity of VEGF-A, at least during early development [60].

In the healthy human adult kidney, VEGFR-1 is expressed in the endothelia of glomeruli, PTC, and pre- and post-glomerular vessels [61,62]. In cultured mouse podocytes under differentiation conditions, mRNA and protein expression of VEGFR-1 have been reported [63]. In mouse glomeruli, VEGFR-1 has been detected in endothelial cells, but not in podocytes under immunoelectron microscopy [64].

### VEGFR-2 (KDR/Flk-1)

VEGFR-2, a high-affinity receptor of VEGF, serves as an important mediator of VEGF-A in inducing mitogenesis, angiogenesis and vascular permeability [65]. VEGFR-2-null mice die *in utero*, primarily due to a lack of vasculogenesis and failure to develop blood islands and organized blood vessels, suggesting a pivotal role for VEGFR-2 in developmental angiogenesis and hematopoiesis [66]. Upon binding of VEGF-A, VEGFR-2 undergoes dimerization and tyrosine phosphorylation, and phosphorylates several proteins in endothelial cells such as phospholipase C-γ, phosphoinositide 3-kinase (PI3K), Ras GTPase-activating protein and Src [67,68]. VEGF-A induces endothelial cell proliferation by activating the Raf-MEK-extracellular signal-regulated (ERK) pathway [69]. Activation of VEGFR-2 prevents apoptosis of endothelial cells through activating PI3K-Akt [36].

In the healthy human adult kidney, VEGFR-2, like VEGFR-1 is expressed in the endothelia of glomeruli, PTC and in pre- and post-glomerular vessels [61,62] and weakly on mesangial cells [61,70]. No expression of VEGFR-2, either at the mRNA or protein level, was observed in cultured mouse podocytes under differentiation conditions [63]. In mouse glomeruli, VEGFR-2 was detected in endothelial cells and also in podocytes (localized to podocyte cell body and foot processes) by immunoelectron microscopy [64].

Considering the dominant production of VEGF-A by podocytes and the localization of VEGFR-2 on glomerular endothelial cells, it is possible that VEGF-A moves across the GBM, opposing the ultrafiltration gradient to move water and solutes from the capillaries into the Bowman's space. Katavetin *et al*. recently demonstrated a kinetic model for VEGF-A transport against glomerular filtration flow [71]. Owing to the size selectivity of the slit diaphragm in the GBM, VEGF-A secreted by podocyte foot processes accumulates in the subslit space and generates a concentration gradient across the GBM, resulting in the diffusion of VEGF-A from podocytes across the GBM to the endothelial cells [71].

### Neuropilin

Neuropilin is implicated in neuronal guidance, and enhances VEGF_165_-mediated chemotaxis [46]. Neuropilin binds to VEGF_165 _in an isoform-specific manner, presenting VEGF_165 _to VEGFR-2 and enhancing the signaling mediated by VEGFR-2 [46]. Neuropilin-1-null mice exhibit embryonic lethality, demonstrating the role of neuropilin-1 in the development of the vascular system [72].

In the healthy human adult kidney, neuropilin-1 is expressed in podocytes, mesangial cells and endothelia of the glomeruli and the afferent arterioles [70,73]. Neuropillin-1 also mediates VEGF-A-induced renal tubular epithelial morphogenesis in mice [74].

### Angiopoietins

The angiopoietin family comprises four structurally related proteins. Ang-1, Ang-2, Ang-3 and Ang-4 [75]. Ang-1 consistently activates and tyrosine-phosphorylates the receptor Tie-2 on endothelial cells, causing enhanced endothelial cell survival and endothelial cell-cell stabilization [76]. Activation of Tie-2 indirectly recruits mesenchymal cells, probably through the action of endothelia-derived paracrine factors, inducing attachment to endothelial tube and differentiation to pericytes, resulting in mature, 'non-leaky' blood vessels [77]. Ang-2, a natural antagonist of Ang-1, loosens the attachment of pericytes, resulting in promotion of sprouting angiogenesis in the presence of VEGF-A [78]. When insufficient angiogenic signals are present, Ang-2 causes endothelial cell death and vessel regression [78]. Ang-2 inhibits activation of Tie-2, and can even specifically block Ang-1-dependent phosphorylation [78]. More recently, activation of Tie-2 by high concentrations of Ang-2 was reported, demonstrating the biological role of Ang-2 not only in antagonizing Ang-1, but also in maintaining a level of Tie-2 activation [79]. Tie-1 is widely expressed by developing endothelia, and it downregulates intracellular signaling triggered by Ang-1-induced phosphorylation of Tie-2 [80]. Mice deficient in Ang-1 die at E12.5, exhibiting some defects in vascular maturation [77], and mice deficient in Ang-2 die 2 weeks after birth, exhibiting some defects in retinal vascularization and lymphatic function [81]. These results indicate the crucial roles of angiopoietins during normal vascular development in the phases of vascular differentiation and maturation. During kidney development, Ang-1, Ang-2, Tie-1 and Tie-2 are strongly expressed, and play pivotal roles in the maturation of glomeruli and renal blood vessels [6].

Ang-1 is expressed in nephrogenic mesenchyme, differentiating tubular epithelia and mature podocytes in humans and rodents [82,83]. Ang-2 is transiently expressed in renal arterial smooth muscle and mesangial cells during development, and is expressed in mature tubules near the *vasa rectae *in mice [84]. Transgenic mice with inducible overexpression of Ang-2 in adulthood, specifically in podocytes, exhibited glomerular endothelial apoptosis and reduction in the levels of VEGF-A and nephrin, accompanied by albuminuria [85], suggesting that upregulation of Ang-2 may destabilize glomerular endothelia and indirectly affect podocytes, leading to deterioration of the glomerular filtration barrier function. Expression of Ang-2 is upregulated by hypoxia, HIF-1α, VEGF-A, angiotensin-II, leptin and glucose in bovine microvascular endothelial cells [86]. Sonic hedgehog upregulates the expression of both Ang-1 and Ang-2 [87]. By contrast, very little is known about the regulatory mechanism of Ang-1.

### Ephrins

The Eph family of receptor tyrosine kinases and their corresponding ligands (ephrins) were originally identified during studies to determine embryonic patterning and neuronal targeting [88]. EphA receptors bind to the ephrinA ligands, and EphB receptors bind to ephrinB ligands, with the exception of EphA4, which binds to both ephrin types [89]. Mice deficient in ephrinB2 or EphB4 die during embryogenesis with severe cardiovascular defects, suggesting the involvement in the primary capillary network remodeling and patterning in embryonic vasculatures [90,91]. The reciprocal expression pattern of ephrinB2 and EphB4 in arterial and venous endothelial cells suggests that they might interact at the arteriovenous interface [92]. EphA2 was observed in tumor-associated vascular endothelial cells, ephrinA1 was detected in tumor and endothelial cells, and soluble EhpA2-Fc exhibits anti-angiogenic and anti-tumor effects, suggesting their involvement in tumor angiogenesis [93].

During mouse kidney development, ephrin-B2 is expressed initially in a subset of podocyte progenitors and subsequently in endothelial cells of the developing glomerulus, while expression of EphB4 was observed in endothelial cells of venous structures [94], suggesting the involvement of interaction between ephrin-B2 and EphB in glomerular microvascular assembly. In the adult mouse kidney, mRNA for EphA1, A2, B4 and ephrin A1 and B1 were found to be strongly expressed [95]. EphB2 was localized in the medullary tubules, and EphB6 in the tubules of the medulla and cortex [96]. By contrast, ephrin-B1 was detected in tubules of the whole mouse nephron [96]. In adult rat glomerulus, ephrin-B1 was found localized at the slit diaphragm, and colocalized with nephrin, a key slit diaphragm protein regulating the glomerular filtration barrier [97]. The role of ephrin-B1 on podocytes in maintaining glomerular filtration barrier function has also been demonstrated [97].

## Role of VEGF-A, VEGFR and other angiogenic growth factors in CKD, both diabetic and non-diabetic

In animal models of non-diabetic CKD including glomerulonephritis, glomerular endothelial cell injuries accompany mesangial alterations or extracapillary lesions. In these models, angiogenic stimuli are required to promote glomerular endothelial repair in association with accelerated resolution of nephritic alterations. In patients in the advanced stages of glomerulonephritis or any other type of renal disorder including hypertension-associated nephrosclerosis and diabetic nephropathy, glomerulosclerosis and tubulointerstitial injuries are observed. In such cases, angiogenic stimuli may also be required, because the rarefaction of PTC and the resulting tubular hypoxia worsen tubulointerstitial injuries. In diabetic nephropathy, especially in the early stages, excessive angiogenic signals are associated with renal alterations and proteinuria in both humans and animals. Therefore, the role of angiogenesis in renal disorders may vary depending on the type and the stage of disease (Table 1).

### VEGF-A and VEGFR

#### In non-diabetic CKD

Proliferation of mesangial cells and expansion of mesangial matrix are central features in various types of glomerular disorders [98,99]. In many forms of glomerulonephritis, severe glomerular injuries are accompanied by glomerular capillary destruction and endothelial cell injuries. Anti-Thy-1 nephritis is one of the widely used models of mesangioproliferative glomerulonephritis [100]. Murine monoclonal 1-22-3 antibody binds to the mesangial cell surface, causing proteinuria and histological alterations, characterized by mesangiolysis followed by monocyte infiltration, mesangial cell proliferation and accumulation of mesangial matrix [101]. In the early phase of rat anti-Thy-1 nephritis following mesangiolysis, reduction in the number of glomerular endothelial cells is observed, followed by initiation of the angiogenic glomerular capillary repair process [102,103]. The essential role of VEGF-A in promoting glomerular capillary repair has been reported in rat anti-Thy-1 nephritis [14,16] and in the rat anti-GBM antibody-induced glomerulonephritis model [15]. In addition, in this model, overexpression of soluble (s)VEGFR-1 accelerated the progression of glomerulosclerosis and interstitial fibrosis accompanied by renal dysfunction and PTC loss [7], further supporting the important role of VEGF-A in the maintenance and repair of glomerular endothelia. The mechanism involved in accelerated glomerulosclerosis in sVEGFR-1-treated nephritic rats might involve indirect effects on mesangial cells due to impaired repair of glomerular endothelial cells in the presence of downregulated VEGF-A.

Progressive loss of endothelia in glomeruli and PTC is observed in advanced stages of CKD with renal dysfunction. Reduced or absent expression of VEGF-A was observed in a variety of advanced CKD in humans [104]. Reduction in renal levels of VEGF-A in association with PTC loss was also observed in animal models, including the thrombotic microangiopathy model [18], in the remnant kidney model induced by 5/6 nephrectomy [17], and in the unilateral ureteral obstruction model [105]. Treatment with VEGF-A resulted in the recovery of PTC number, associated with amelioration of tubulointerstitial injuries in the thrombotic microangiopathy and the remnant kidney models [17,18].

VEGFR-1 was observed in mesangial cells in biopsies taken from patients with mesangioproliferative glomerulonephritis [70]. The monocyte-macrophage lineage cells expressed VEGFR-1, and a VEGFR-1 neutralizing monoclonal antibody significantly suppressed VEGF-A-induced migration of the monocytes, suggesting the involvement of VEGFR-1 in monocyte infiltration [106].

Pre-eclampsia remains a major cause of human maternal and fetal morbidity and mortality, affecting about 5% of pregnancies [107]. Although the precise mechanism of this disease remains unclear, dysfunction in the maternal vascular endothelium plays a crucial role in the development of pre-eclampsia, resulting in hypertension and proteinuria [108]. Previous studies have suggested that inadequate placental development might lead to placental ischemia, resulting in the release of placental factors that damage the maternal vascular endothelium [109]. Recent reports have demonstrated the involvement of angiogenesis-associated factors in pre-eclampsia. Alterations in the serum level of VEGF-A and decreases in the level of circulating PlGF in patients with pre-eclampsia have been reported [110-112]. The levels of sVEGFR-1, a naturally occurring antagonist of VEGF-A, in amniotic fluid were increased in women with pre-eclampsia [113], and increased production of sVEGFR-1 by cytotrophoblasts isolated from women with pre-eclampsia has been reported [114]. More recently, increases in the level of circulating sVEGFR-1 in women with pre-eclampsia have been reported [112,115]. In patients with CKD, excess circulating sVEGFR-1 levels were associated with endothelial dysfunction, suggesting that increased sVEGFR-1 levels may predict cardiovascular risk in CKD [116].

Similarly, VEGFR-2 was found in mesangial cells in biopsies taken from patients with mesangioproliferative glomerulonephritis [70]. In patients with focal segmental glomerulosclerosis, tubulointerstitial levels of neuropilin-2 were increased, and these correlated with progressive decline in renal function [117].

### In diabetic nephropathy

Diabetic nephropathy is a complication that occurs in 30-40% of patients with type 2 diabetes, and is the most common pathological disorder predisposing to ESRD in Japan and in western countries [118]. Early alterations in diabetic nephropathy include glomerular hyperfiltration, glomerular and tubular epithelial hypertrophy, and development of microalbuminuria [119]. These early alterations are followed by the development of GBM thickening, accumulation of extracellular matrix (ECM) components in mesangium and interstitium, and increase in excretion of urinary albumin, eventually leading to glomerulosclerosis and progressive loss of renal function [99,120]. Involvement of angiotensin II, IGF-I and TGF-β^1 ^in the development of diabetic nephropathy has been reported [121,122].

In patients with type 1 or 2 diabetic nephropathy, histological alterations with abnormal blood vessels in the glomeruli have been reported [123-125]. The abnormal vessels were seen in the glomerular capillary area, Bowman's capsule and the glomerular vascular pole [123,124,126]. Previous studies with rodent models of type 1 and 2 diabetes have demonstrated that the increased glomerular filtration surface in diabetic nephropathy results from the formation of new glomerular capillaries, as well as a slight elongation of the pre-existing capillaries [24,127], analogous to the changes observed in pathologic diabetic retinopathy.

Protein and mRNA level of VEGF-A and VEGFR-2 were found to be upregulated in the early and late stages of rat experimental diabetic nephropathy [10,128]. In patients with diabetic nephropathy, plasma [129] and urinary [130] levels of VEGF-A were elevated. In renal biopsies from patients with diabetic nephropathy, glomerular levels of VEGF-A were either increased [124,131] or decreased [132], and tubulointerstitial levels of VEGF-A were reduced [133]. These discrepancies in the glomerular levels of VEGF-A may be attributable to the stage of diabetic nephropathy; that is, reduced renal VEGF-A levels in the advanced stages of disease.

In cultured podocytes, high levels of glucose, TGF-β1 and angiotensin-II induce expression of VEGF-A [134]. VEGF-A, produced by podocytes, acts through VEGFR-1 also located on podocytes and activates PI3K, leading to increased production of α3(IV) collagen, a principal component of the GBM, suggesting an autocrine role for VEGF-A [63]. These results suggest a role for TGF-β1-induced endogenous VEGF-A in podocyte dysfunction, GBM thickening and alteration of the glomerular filtration barrier in diabetic nephropathy. VEGF-A also decreases intracellular calcium concentration, and exerts protective effects against cytotoxic insult [135].

To address the discrepancies in function of VEGF-A in non-diabetic CKD and diabetic nephropathy, Nakagawa *et al*. proposed the hypothesis of 'uncoupling of the VEGF-endothelial NO axis' in diabetic nephropathy [136]. Endothelial dysfunction induced by hyperglycemia or other factors may underlie the pathogenic mechanisms of an elevated VEGF state. VEGF-A normally stimulates endothelial NO release and acts as a trophic factor for vascular endothelium in concert with elevated NO levels. The increased NO derived from the endothelial cells prevents excessive proliferation of endothelial and vascular smooth muscle cells and infiltration of monocytes/macrophages. When NO bioavailability is reduced, as in diabetes, elevated levels of VEGF-A lead to excessive endothelial cell proliferation, stimulation of macrophage infiltration and activation of vascular smooth muscle cells. Consistent with this hypothesis, diabetes induced in endothelial NO-deficient mice resulted in clinical and histological features identical to human diabetic nephropathy [137]. The role of VEGF-A in promoting macrophage infiltration through VEGFR-1 and the inhibitory effects of NO on VEGF-A-induced macrophage infiltration were also demonstrated in diabetic endothelial nitric oxide synthase knockout mice [138].

### Angiopoietins

#### In non-diabetic CKD

Futrakul *et al*. reported reduction in circulating levels of VEGF-A and Ang-1, and elevation of Ang-2 levels in patients with CKD who had moderate to severe renal dysfunction [139]. In a mouse model of folic acid-induced nephrotoxicity, Ang-1 was detected in renal arterial walls and in injured cortical distal tubules [11]. In a mouse anti-GBM nephritis model, the glomerular level of Ang-1 was reduced and Ang-2 was increased, in association with glomerular endothelial cell apoptosis [12]. In a mouse model of unilateral ureteral obstruction, reduction in renal Ang-1 level was observed. Ang-1 gene therapy in this mouse model, using an adenoviral vector encoding a chimeric form of Ang-1 called cartilage oligomeric matrix protein (COMP)-Ang-1, resulted in preservation of PTC and reductions in monocyte/macrophage infiltration and interstitial fibrosis [22]. In a mouse folic acid-induced nephrotoxicity model, adenoviral delivery of Ang-1 resulted in stabilization of PTC, but also induced inflammatory and fibrotic responses in the kidney [140], suggesting that Ang-1 supplementation can have variable outcomes on renal injuries, depending on the disease model. Some recent studies demonstrated that Ang-1 could have pro-inflammatory effects. Murine macrophages expressed Tie-1 and Tie-2 *in vitro *[140], neutrophils also expressed Tie-2 [141], and treatment of aortic rings with Ang-1 upregulated chemokines [142].

In a rat angiotensin-II infusion model, renal levels of VEGF-A, Ang-1 and Ang-2 were increased via angiotensin-II type 1 and 2 receptors [143]. In the same rat model, we observed an increase in the renal Ang-1:Ang-2 ratio, potentially counteracting VEGF-A-induced vascular permeability after infusion of angiotensin-II [144]. In addition, murine macrophages express Ang-2, and this has been implicated in the aggravation of inflammation [145]. Pharmacological blockade of the renin-angiotensin system ameliorated the pathological changes seen in rat anti-Thy-1 nephritis [146]; however, infusion of angiotensin-II before the induction of rat anti-Thy-1 nephritis unexpectedly resulted in accelerated resolution of nephritic alterations in the early stages [147]. In the same rat model, we observed accelerated repair of glomerular endothelium accompanied by renal induction of VEGF-A signaling and increase in the Ang-1:Ang-2 ratio [148].

We have observed elevated serum Ang-2 level in healthy pregnant women compared with non-pregnant women, and significant suppression of circulating Ang-2 in women with pre-eclampsia [149]. The serum Ang-2 concentrations inversely correlated with proteinuria in women with pre-eclampsia [149]. These findings suggest an essential role for Ang-2 in placental angiogenesis during pregnancy.

#### In diabetic nephropathy

In patients with type 2 diabetes, elevated circulating levels of Ang-2 and VEGF-A were observed [150]. In a rat model of type 1 diabetic nephropathy, decreased renal level of Ang-1 and elevation of Ang-2 were observed at 8 weeks after the induction of diabetes and Ang-2 was observed in glomerular endothelia and podocytes [13]. We also observed upregulation of renal Ang-2 levels and decreased Ang-1/Ang-2 ratio accompanied by glomerular monocyte/macrophage infiltration in mouse type 1 and 2 diabetic nephropathy models [25-27]. In a mouse model of obese type 2 diabetes, Ang-1 gene therapy using adenoviral vector encoding COMP-Ang-1 resulted in the reduction in albuminuria, amelioration of mesangial expansion and podocyte injuries accompanied by reduced monocyte/macrophage infiltration and downregulation of chemokines and adhesion molecules [151].

### Other angiogenic factors

In a mouse model of ischemia-reperfusion injury (IRI), renal expression of EphA2 mRNA was markedly increased and EphA2 protein was detected in distal tubules [95]. Interactions between EphA2 and its ephrin ligands may be involved in cytoskeletal repair of tubular epithelial cells in renal IRI.

PlGF, a member of VEGF family, stimulates pathological angiogenesis through activating VEGFR-1 without any influence on physiological processes [152]. In the early stages (1 or 2 weeks) of the rat remnant kidney model, renal levels of PlGF were elevated in association with enhanced angiogenic response [153]. Whether VEGF-A [17] or PlGF play principal roles in the angiogenic repair process after injuries induced by renal mass ablation needs further investigation.

## Role of angiogenesis inhibitors in CKD, both diabetic and non-diabetic

Although findings about the biological role of angiogenesis inhibitors in CKD are relatively limited, therapeutic effects have been reported for angiogenesis inhibitors in experimental diabetic nephropathy models. Angiogenesis inhibitors exhibit therapeutic effects on diabetic nephropathy by anti-angiogenic and anti-inflammatory mechanisms, as inhibition of excessive neovessels may reduce the infiltration of inflammatory cells, and downregulation of VEGF-A may ameliorate vascular permeability. However, considering the reduction in PTC density associated with tubular hypoxia and tubulointerstitial injuries in advanced CKD, use of angiogenesis inhibitors may need careful consideration.

### Angiogenesis inhibitors in non-diabetic CKD

#### Angiostatin

Angiostatin, a proteolytic fragment of plasminogen, potently blocks neovascularization, tumor growth and metastasis [154]. In healthy rat kidney, angiostatin is not expressed, but its level was enhanced in the first 3 days after renal IRI, and remained detectable until 35 days after induction of ischemia-reperfusion (I/R) [155]. Renal angiostatin generation after acute renal failure induced by I/R may modulate postischemic renal capillary density and thereby influence chronic renal dysfunction.

In a rat remnant kidney model, pretreatment with adenoassociated viral vectors encoding angiostatin resulted in reduction in PTC density and urinary NO levels [156]; however, in another study, interstitial fibrosis and monocyte infiltration was ameliorated by treatment with angiostatin [156]. Therefore, angiostatin may exert therapeutic effects in CKD through its anti-inflammatory actions, but potential adverse events (such as reducing PTC through its anti-angiogenic effects, thus leading to the acceleration of tubular hypoxia/ischemia) have to be considered.

### Angiogenesis inhibitors in diabetic nephropathy

#### Anti-VEGF-A therapies

As described in the previous section, renal levels of VEGF-A and VEGFR-2 were increased in both early and late stages of rat experimental diabetic nephropathy [10,128]. Animal studies using monoclonal anti-VEGF-A antibodies have demonstrated the involvement of VEGF-A in early glomerular hypertrophy and mesangial matrix accumulation during the progressive stages of diabetic nephropathy [19,20]. In addition, SU5416, a pan-VEGF receptor tyrosine kinase inhibitor, reduced albuminuria in the type 2 diabetes db/db mouse model [21]. Podocyte-specific, doxycycline-inducible overexpression of sVEGFR-1 in adult mice ameliorated diabetic glomerular injuries, further implicating the involvement of VEGF-A in the pathogenesis of diabetic nephropathy [64].

#### Tumstatin

Type IV collagen is expressed as six distinct α-chains (α1 to α6), assembles into triple helices, and forms organized networks. The α-chains consist of an N-terminal 7 S domain, a central collagenous triple helical domain and a C-terminal globular NC1 domain. Although the α1(IV) and α2(IV) chains are ubiquitously distributed in human basement membranes, distribution of the α3(IV) chain is limited to certain basement membranes, such as GBM, several basement membranes of the cochlea, and the alveolar capillary basement membrane [157].

α3(IV)NC1 (also known as tumstatin, a term derived from its unique property of causing 'tumor-stasis'), inhibits angiogenesis and tumor growth by suppression of endothelial cell proliferation and tube formation [158-160]. Representative matrix-derived angiogenesis inhibitors are listed in Table 2. Tumstatin exerts its anti-angiogenic effects by binding to αvβ3 integrin in an Arg-Gly-Asp (RGD)-independent manner [161,162].

**Table 2:** Endogenous basement membrane-derived angiogenesis inhibitors

Angiogenesis inhibitor	Parent protein	Domain	Receptors	Inhibitory activities on EC
Endostatin	α1 chain of type XVIII collagen	NC1	Glypicans, flk-1, α 5β1-integrin	Proliferation, migration, tube formation, survival, tumor growth
Restin	α1 chain of type XV collagen	NC10	Unknown	Proliferation, migration, tumor growth
Tumstatin	α3 chain of type IV collagen	NC1	αvβ3-integrin	Proliferation, tube formation, survival, protein translation, tumor growth
Arresten	α1 chain of type IV collagen	NC1	α1β1-integrin	Proliferation, migration, tube formation, survival, tumor growth
Canstatin	α2 chain of type IV collagen	NC1	α3β1-integrin	Proliferation, migration, tube formation, survival, tumor growth
α6(IV)NC1	α6 chain of type IV collagen	NC1	Unknown	Proliferation
Endorepellin	Perlecan	Domain V	α 2β1-integrin	Migration, tube formation, survival, blood vessel growth *in vivo*

EC, endothelial cell. ECM, extracellular matrix.

Goodpasture syndrome is an autoimmune disease characterized by pulmonary hemorrhage and/or rapidly progressive glomerulonephritis. The most probable disease-related pathogenic autoepitope was identified in the tumstatin sequence [163], within the N-terminal 40 amino acids [164]. Using deletion mutants of tumstatin and synthetic peptides, the domain possessing anti-angiogenic properties was found to be confined to the T3 (69-88 amino acids), T7 (74-98 amino acids) and T8 peptide regions of tumstatin, outside the Goodpasture epitope [165,166].

Apoptosis is regulated in part at the level of protein synthesis, and is generally associated with inhibition of cap-dependent protein translation. Tumstatin peptides exhibit endothelial cell-specific inhibitory effects on cap-dependent translation by negatively regulating activation of focal adhesion kinase (FAK), PI3K, Akt, mTOR and eukaryotic initiation factor 4E binding protein 1 [167]. These inhibitory effects of tumstatin peptides were not observed in β3-integrin-deficient endothelial cells, implying an interaction of tumstatin peptides with αvβ3 integrin-induced negative regulatory signals counteracting growth factor-initiated cell survival signals [167].

Because histological 'angiogenic' abnormal vessels in the glomerular capillary area, Bowman's capsule and the glomerular vascular pole are observed in the early stages of diabetic nephropathy in both humans and rodents [24,123,124,127], and the involvement of VEGF-A was demonstrated in the progression of experimental diabetic nephropathy [19,20], we hypothesized that anti-angiogenic factors might inhibit the progression of diabetic nephropathy. To test this hypothesis, we examined the therapeutic effect of tumstatin in the early stages of diabetic nephropathy in the streptozotocin (STZ)-induced mouse type 1 diabetes model [25]. Treatment with the tumstatin T8 peptide did not affect hyperglycemia, but significantly suppressed renal hypertrophy, glomerular hypertrophy/hyperfiltration and albuminuria, and histological alterations such as mesangial expansion, increase in glomerular capillary number and monocyte/macrophage infiltration in diabetic animals. The tumstatin (T8) peptide significantly suppressed the STZ-induced increase in VEGF-A and VEGFR-2 in the renal cortex, suggesting that the therapeutic effect of the tumstatin (T8) peptide in early diabetic nephropathy may be at least partly attributable to the inhibition of excessive activation of VEGF-A signaling, analogous to previous studies using neutralizing anti-VEGF-A antibodies [19,20]. The tumstatin (T8) peptide treatment did not affect the renal levels of Ang1, but suppressed Ang-2 increase in diabetic mice, leading to elevation of the Ang-1:Ang-2 ratio [25]. The marked increase in Ang-2 over Ang-1 in diabetic mice suggests that there is a pro-angiogenic environment, considering the simultaneous upregulation of VEGF-A and the unstable, 'leaky' condition of capillaries, possibly associated with inflammatory responses such as monocyte/macrophage recruitment.

Nephrin is a podocyte protein crucial for maintenance of an intact glomerular filtration barrier [168]. In diabetic nephropathy, the protein level of nephrin decreases in association with podocyte injury and proteinuria, and tumstatin restored the level of nephrin, possibly associated with its anti-albuminuric effects [25]. The indirect influence of glomerular endothelial cells toward podocytes possibly mediated via secreted factors or alteration on matrix microenvironment, might be involved in this mechanism.

Collectively, these results suggest that the tumstatin peptide may have therapeutic potential in human diabetic nephropathy, in addition to its potent anti-tumor effect.

#### Endostatin

Endostatin, a 20 kDa C-terminal NC1 domain of type XVIII collagen, possesses potent anti-angiogenic activity [169]. Endostatin was initially isolated from the conditioned medium of murine hemangioendothelioma cells. Endostatin inhibits endothelial cell proliferation, migration and tube formation *in vitro *[170,171], and possesses potent inhibitory effects on tumor growth *in vivo*. The inhibitory effects of endostatin on the expression of VEGF in tumor cells and on vascular permeability have been reported [172]. The therapeutic potential of endostatin in non-neoplastic disorders with involvement of angiogenic processes (for example, rheumatoid arthritis, proliferative diabetic retinopathy and peritoneal sclerosis) has also been reported [173-175]. The anti-angiogenic activity of endostatin was confined to the peptide region within the N-terminal domain [176].

Human endostatin interacts with α5β1 integrin, leading to the inhibition of the activation of FAK and subsequent inhibition of mitogen-activated protein kinases (MAPKs) including ERK-1/2 and p38MAPK, resulting in inhibition of endothelial cell migration [177]. In addition, glypicans, a form of heparan sulfate glycosaminoglycans, bind to endostatin, and this interaction is important for mediating the anti-angiogenic activities of endostatin *in vitro *[178].

We examined the therapeutic effects of endostatin in the early stages of diabetic nephropathy utilizing the STZ-induced mouse type 1 diabetes model [26]. Although treatment with a synthetic endostatin peptide encompassing the 1-27 amino acid portion of human endostatin did not affect hyperglycemia in this model, there was significant suppression of renal hypertrophy, glomerular hypertrophy/hyperfiltration and albuminuria, along with histological alterations [26]. The endostatin peptide significantly suppressed the STZ-induced increase in VEGF-A, VEGFR-2, Ang-2, TGF-β1, IL-6 and monocyte chemotactic protein (MCP)-1 in renal cortex and restored the level of nephrin [26]. Because a central involvement of α5β1 integrin in mediating the anti-angiogenic capacity of endostatin has been reported [177], we studied the localization of this cell surface receptor in diabetic mice. Although expression of α5β1 integrin was faintly present in the glomeruli of non-diabetic control mice, increased expression of α5β1 integrin colocalized with CD31 was observed in the glomeruli of diabetic mice, as detected by double immunofluorescence [26]. These results suggest that the primary action of endostatin on glomerular endothelial cells mediated via α5β1 integrin in diabetic mice. Therefore, we speculate that endostatin indirectly affects podocytes leading to decreased expression of VEGF-A in diabetic mice.

#### NM-3

2-(8-hydroxy-6-methoxy-1-oxo-1H-2-benzopyran-3-yl) propionic acid (NM-3) is a synthetic derivative of cytogenin, a natural compound isolated from filtrates of *Streptoverticillium eurocidicum *cultures [179]. NM-3 potently inhibits endothelial cell proliferation, migration, sprouting and tube formation *in vitro *and tumor growth *in vivo *[180]. Moreover, NM-3 suppresses endothelial cell migration induced by VEGF-A, and exerts inhibitory effects on angiogenesis in VEGF-A-secreting malignant tumors [180]. A phase I clinical study of NM-3 in patients with cancer has demonstrated that NM-3 has high oral bioavailability and is a well-tolerated drug in humans [181].

We examined the therapeutic effect of NM-3 on long-term renal alterations in db/db mice, a model of obese type 2 diabetes [27]. Treatment with NM-3 was initiated at 8 weeks of age in db/db mice exhibiting hyperglycemia and was continued until 16 weeks. Although treatment with NM-3 did not affected either obesity or hyperglycemia, significant suppression of renal hypertrophy, glomerular hypertrophy/hyperfiltration and albuminuria, as well as histological alterations were observed in diabetic animals [27]. NM-3 significantly suppressed the increase in VEGF-A, Ang-2, TGF-β1 and MCP-1 seen in the renal cortex, and restored the level of nephrin in db/db mice [27]. The direct inhibitory effect of NM-3 on phosphorylation of VEGFR-2 in glomerular endothelial cells remains to be elucidated. However, inhibitory effects of NM-3 on renal levels of VEGF-A suggest a potential inhibitory effect of NM-3 on the phosphorylation of VEGFR-2 in glomerular endothelial cells, resulting in the observed therapeutic effects of NM-3 in diabetic mice. *In vitro *analysis further demonstrated the direct inhibitory effect of NM-3 on the synthesis of TGF-β in mesangial cells and the production of VEGF-A in podocytes induced by high glucose [27]. These results demonstrate the direct effect of NM-3 on mesangial cells and podocytes under high glucose conditions, in addition to its known effects on endothelial cells, and the efficacy of angiogenesis inhibitors on long-term renal alterations in obese type 2 diabetes.

#### Angiostatin

Angiostatin inhibits retinal neovascularization and reduces retinal vascular permeability in diabetic retinopathy. Zhang *et al*. examined the potential involvement of angiostatin in diabetic nephropathy using an STZ-induced type 1 diabetes rat model [28]. Angiostatin levels were dramatically decreased in the kidneys of control diabetic rats, and adenovirus-mediated delivery of angiostatin significantly ameliorated albuminuria and glomerular hypertrophy in diabetic rats. Inhibitory effects of angiostatin treatment on VEGF-A and TGF-β1 in diabetic kidneys were also observed [28]. In cell culture analysis, angiostatin blocked overexpression of VEGF-A and TGF-β1 induced by high levels of glucose, while increasing the levels of pigment epithelium-derived factor (PEDF), an endogenous inhibitor of diabetic nephropathy. Similar to NM-3, angiostatin produced direct effects on mesangial cells and endothelial cells, as well as therapeutic effects on diabetic nephropathy.

#### PEDF

PEDF is a potent inhibitor of angiogenesis, and decreased ocular levels of PEDF are associated with diabetic retinopathy. Wang *et al*. have demonstrated that renal expression of PEDF was decreased in the STZ-induced type 1 diabetic rat model, and high levels of glucose significantly decreased PEDF secretion in primary human mesangial cells (HMC) *in vitro *[182]. PEDF blocked the overexpression of TGF-β and fibronectin induced by high glucose levels in primary HMC [182]. Wang *et al*. also showed that adenoviral delivery of PEDF into the kidney significantly ameliorated microalbuminuria in the early stages of diabetes by suppressing TGF-β1 and connective tissue growth factor and enhancing MMP-2, thus leading to reduced production of ECM proteins in the diabetic kidney [29]. They further demonstrated the anti-inflammatory action of PEDF therapy in the same model; PEDF regulated renal levels of intracellular adhesion molecule-1, MCP-1, TNF-β and VEGF-A, and it also inhibited activation of the transcription factors nuclear factor kappa B and HIF-1 in mesangial cells [183].

#### Vasohibin-1

**Figure 1:**
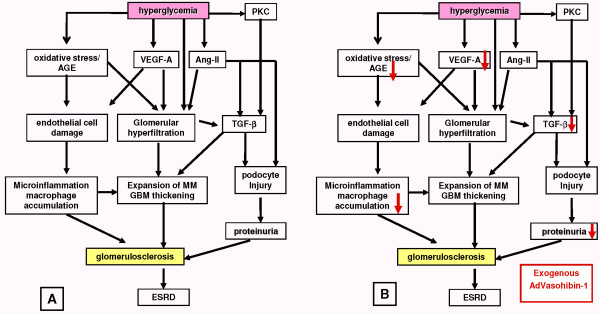
**Mechanisms of action of vasohibin (VASH)-1 in diabetic nephropathy**. **(A) **Mechanisms involved in the progression of diabetic nephropathy. **(B) **Exogenous VASH-1 suppresses renal alterations in the experimental mouse type 1 diabetes model through anti-angiogenic, anti-inflammatory and anti-fibrotic effects and also through protecting podocytes. VASH-1 suppresses excessive activation of VEGF-A signaling on glomerular endothelial cells, and also exerts direct effects on mesangial cells and podocytes in diabetic environments [30]. AGE = advanced glycation end-product; Ang-II = angiotensin-II; ESRD = end stage renal disease; GBM = glomerular basement membrane; MM = mesangial matrix; PKC = protein kinase C; TGF-β = transforming growth factor-β.

Vasohibin (VASH)-1, an endogenous angiogenesis inhibitor, was identified in a microarray analysis assessing genes upregulated by VEGF-A in endothelial cells [184]. VASH-1 serves as an endothelial cell-derived negative feedback regulator of angiogenesis, and its therapeutic efficacies on tumor growth, atherosclerosis and proliferative retinopathy models have been reported [184-186]. The potential of VASH-1 as a biomarker of disorders associated with angiogenesis was demonstrated for endometrial cancer and rheumatoid arthritis [187,188]. We recently reported the therapeutic effects of VASH-1 in the mouse STZ-induced type 1 diabetic nephropathy model [30]. Mice were given intravenous injections of adenoviral vectors encoding VASH-1 (AdhVASH-1) every 2 weeks. Treatment with AdhVASH-1 resulted in sustained increase in the circulating levels of VASH-1, and significantly suppressed renal and glomerular hypertrophy, glomerular hyperfiltration, albuminuria, increase in the CD31+ glomerular endothelial area and F4/80+ monocyte/macrophage infiltration, and accumulation of type IV collagen and mesangial matrix compared with AdLacZ-treated diabetic mice at 4 weeks after initiating treatment [30]. Interestingly, VASH-1 treatment did not affect the microvascular density of PTC in diabetic animals. Increase in the renal levels of TGF-β1, MCP-1 and RAGE (receptor for advanced glycation end products) in diabetic animals was significantly suppressed by AdhVASH-1. VASH-1 further suppressed increase in TGF-β1, MCP-1 and RAGE induced by high glucose levels in cultured mouse mesangial cells. Increased phosphorylation of VEGFR2 was suppressed in the kidneys of AdhVASH-1-treated diabetic animals and in cultured glomerular endothelial cells [30]. Endogenous mouse VASH-1 was localized to the mesangial and endothelial areas in glomeruli of diabetic mice. These results suggest therapeutic potential for VASH-1 in treating early diabetic nephropathy thought to be mediated via glomerular endothelial and mesangial cells (Figure 1). We recently observed potential protective effects of VASH-1 on glomerular podocytes, possibly associated with anti-albuminuric effect (manuscript in submission).

**Figure 2:**
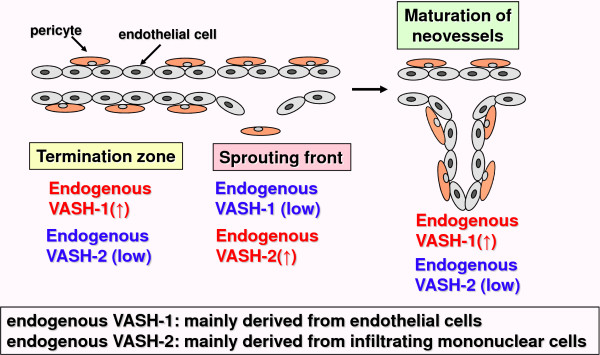
**Regulation of angiogenic process by endogenous VASH-1 and VASH-2**. Levels of endogenous VASH-1 are decreased in proliferating endothelial cells at the sprouting front, but elevated in non-proliferating endothelial cells at the termination zone of angiogenesis, presumably serving to halt angiogenesis (189). Levels of endogenous VASH-2, a homologue of VASH-1 that is mainly derived from infiltrating mononuclear cells, are elevated at the sprouting front, but low at the termination zone of angiogenesis, serving to induce angiogenesis (189).

Levels of endogenousVASH-1 are decreased in proliferating endothelial cells at the sprouting front, but are elevated in non-proliferating endothelial cells at the termination zone of angiogenesis, presumably serving to halt angiogenesis [189]. VASH-2, a homologue of VASH-1, was identified subsequently, and is mainly derived from infiltrating mononuclear cells mobilized from bone marrow, which infiltrate the sprouting front. Levels of endogenous VASH-2 are elevated at the sprouting front, but are low at the termination zone of angiogenesis, presumably serving to induce angiogenesis [189]. Exogenous VASH-1 inhibited angiogenesis at the sprouting front, where endogenous VASH-1 was scarce, but did not influence vascularity in the termination zone, where endogenous VASH-1 was high [189]. Exogenous VASH-2 prevented the termination of angiogenesis, and increased vascularity in the termination zone [189]. Angiogenesis was persistent in the termination zone in VASH-1 knockout mice, but was deficient at the sprouting front in VASH-2 knockout mice [189]. Regulation of the balance between VASH-1 and VASH-2 may be involved in angiogenesis and vascular maturation, and may be involved in the progression and the control of CKD (Figure 2).

### Potential problems in using anti-angiogenic therapies in patients with CKD

The potential adverse events of bevacizumab, a humanized monoclonal antibody against VEGF-A have been reported in patients with cancer, with administration of the drug resulting in thrombotic microangiopathy [53]. The lack of such histological alterations in previous experimental diabetic nephropathy models treated with anti-VEGF antibodies or SU5416 [19-21], and amelioration of diabetic glomerular alterations by inducible podocyte-specific overexpression of sVEGFR-1 in adult mice [64] suggest that anti-VEGF-A therapy might not be detrimental for patients with diabetic nephropathy. In fact, systemic delivery of sVEGFR-1 resulted in the protection of podocytes and a reduction in albuminuria, but exacerbated tubulointerstitial injuries accompanied by PTC loss in db/db mice [190]. Although tumstatin, endostatin, NM-3 and VASH-1 suppress overactivation of VEGFR-2, they do not serve as specific inhibitors of VEGF-A signaling, unlike bevacizumab or other specific anti-VEGF-A therapeutics. Because PTC loss, chronic hypoxia or tubulointerstitial injuries are involved in the progression of CKD [191], careful evaluation of patients' long-term outcome after anti-angiogenic therapeutics is required.

In the advanced stages of CKD including diabetic nephropathy, anti-angiogenic reagents may impair neovessel formation, leading to deterioration of macrovascular complications such as myocardial infarction and limb ischemia. Thus, considering the involvement of angiogenesis in atherosclerosis., anti-angiogenic therapeutics might be tolerable or even therapeutic for atherosclerotic conditions. In fact, the therapeutic effects of VASH-1 in preventing neointimal formation have been reported [185]. In addition, VASH-1 does not impair healthy blood or lymphatic vessels in mice [192], suggesting relative safety of this factor in treating patients with diabetic and non-diabetic CKD. Regarding the question of using adenoviral vectors to treat patients with diabetes, potential adverse events such as non-specific inflammatory reactions and the replication of adenoviruses *in vivo *must be avoided, and further assessments on the safety of this strategy are required.

## Conclusion

We have looked at recent advances on the involvement of angiogenic and antiangiogenic factors in CKD and potential experimental approaches using angiogenesis inhibitors to treat CKD, including diabetic nephropathy. Based on the mechanistic insight into the involvement of angiogenesis in CKD, use of novel therapeutic reagents in patients with CKD in clinical practice is expected in the near future, leading to the eventual reduction in the number of patients developing ESRD.

## Competing interests

YM and HM have submitted a patent application for treating diabetic nephropathy and peritoneal sclerosis by vasohibin-1.

## Authors' contributions

YM carried out experimental studies and drafted the manuscript. HM participated in the design of experiments and helped to draft the manuscript. All authors read and approved the final manuscript.
